# Supplementary material for: Anti-Fibrotic and Anti-Angiogenic Activities of Osbeckia octandra Leaf Extracts in Thioacetamide-Induced Experimental Liver Cirrhosis
Source: Molecules. 2021 Aug 10;26(16):4836. doi: 10.3390/molecules26164836 (PMC8401385; doi:10.3390/molecules26164836)
Supplement: Supplementary file 1 [file molecules-26-04836-s001.zip › molecules-1299786 Supplymentary Figures, Tables and Legends-F.pdf]

Table S1: Qualitative analysis of phytochemicals for different extracts of *O.octandra* leaves

**Chemical Test**	**BLE**	**SLE**	**MLE**	**HLE**
Phenols and tannins	+	+	+	+
Flavonoids	+	+	+	+
Steroids	+	+	+	+
Alkaloids	+	+	+	+
Terpenoids	+	+	nd	nd

Presence of relevant phytochemical marked as + and absence marked as nd

Table S2: Effect of CLS (crude leaves suspension) on body weight, organ weight and serum parameters.

**Parameter (mg/kg BW)**	**Con**	**250**	**500**	**1,000**	**p value**
BW gain (g)	96.80 ± 5.18^{ab}	108.00 ± 2.88^{a}	92.85 ± 5.24^{ab}	80.00 ± 3.62^{b}	0.003
Liver Index (%)	12.03 ± 0.16^{a}	11.60 ± 0.28^{a}	11.76 ± 0.19^{a}	11.83 ± 0.19^{a}	0.474
Kidney weight(g)	1.07 ± 0.02^{b}	1.26 ± 0.04^{ab}	1.13 ± 0.03^{ab}	1.32 ± 0.08^{a}	0.009
Spleen weight (g)	1.05 ± 0.27^{b}	1.08 ± 0.32^{b}	1.55 ± 0.07^{a}	1.69 ± 0.11^{a}	0.000
Lung weight (g)	1.63 ± 0.11^{a}	1.47 ± 0.12^{a}	1.37 ± 0.05^{a}	1.31 ± 0.07^{a}	0.112
ALT (U/L)	102.56 ± 4.33^{a}	94.40 ± 2.27^{a}	97.18 ± 5.28^{a}	97.48 ± 4.26^{a}	0.588
AST (U/L)	119.40 ± 2.69^{a}	92.40 ± 6.28^{a}	102.40 ± 9.37^{a}	109.60 ± 8.45^{a}	0.094

Values are expressed in mean ± SEM, n=5, Statistically significant from control $p<0.05$. Different letters denote significant difference at ($p<0.05$)

Table S3: Effect size analysis

**Parameter**	**Group comparisons**	**Cohen's d_s**	**Hedges's g_s**	**Eta squared**	**Omega squared**
Body Weight Gain	HC vs DC	-18.553	-16.758	0.944	0.990
	DC vs CLS	-17.274	-15.602	0.634	0.338
	DC vs BLE	-09.547	-08.623	0.771	0.068
	DC vs SLE	-06.092	-05.502	0.992	0.962
	DC vs MLE	-01.272	-01.148	0.453	0.768
	DC vs HLE	-00.472	-00.427	0.945	0.741
Liver Index	HC vs DC	12.185	11.006	0.951	0.561
	DC vs CLS	09.924	08.963	0.583	2.059
	DC vs BLE	08.431	07.615	0.954	0.779
	DC vs SLE	07.319	06.611	0.904	0.561
	DC vs MLE	00.920	00.831	0.679	-0.216
	DC vs HLE	-16.532	-14.932	0.976	0.881
Serum AST	HC vs DC	13.251	11.968	0.969	0.849
	DC vs CLS	10.360	09.357	0.982	0.914
	DC vs BLE	07.402	06.686	0.998	0.990
	DC vs SLE	-16.532	-14.932	0.976	0.881
ALT	HC vs DC	-18.038	-16.292	0.742	-0.026

	DC vs CLS	21.344	19.278	0.944	0.735
	DC vs BLE	19.963	18.031	0.154	-1.291
	DC vs SLE	16.180	14.614	0.951	-0.027
ALP	HC vs DC	-22.658	-20.465	0.966	0.836
	DC vs CLS	21.851	19.737	0.923	0.641
	DC vs BLE	09.086	08.207	0.983	0.916
	DC vs SLE	02.600	02.349	0.667	-0.250
Image J analysis	HC vs DC	-08.997	-08.127	0.375	-0.923
	DC vs CLS	08.036	07.258	0.984	0.921
	DC vs BLE	06.710	06.061	0.908	0.577
	DC vs SLE	04.475	04.042	0.074	-1.403
	DC vs MLE	00.013	00.012	0.958	0.799
	DC vs HLE	-00.663	-00.599	0.679	-0.216
Tnf-α1 Expression	HC vs DC	01.610	01.454	0.444	-0.087
	DC vs CLS	-00.643	-00.580	0.103	-0.549
	DC vs BLE	-01.345	-01.214	0.667	0.286
	DC vs SLE	-00.775	-00.700	0.615	0.194
α-Sma Expression	HC vs DC	09.228	08.335	0.986	0.943
	DC vs CLS	-02.160	-01.951	0.141	-0.848
	DC vs BLE	-03.391	-03.063	0.971	0.887
	DC vs SLE	-01.499	-01.354	0.998	0.994
Tgf-β Expression	HC vs DC	06.928	06.258	0.151	-0.324
	DC vs CLS	-04.893	-04.420	0.056	-0.436
	DC vs BLE	-06.943	-06.271	0.906	0.817
	DC vs SLE	-06.310	-05.699	0.059	-0.432
Vegf-R2 Expression	HC vs DC	06.352	05.737	0.541	0.023
	DC vs CLS	-06.183	-05.585	0.113	-0.536
	DC vs BLE	-03.592	-03.245	0.457	-0.068
	DC vs SLE	-01.806	-01.631	0.608	0.181

Interpretation of effect: Cohen's d_s and Hedges'g_s: 0.01; very small, 0.2; small, 0.5; medium; 0.8 large, 1.2; very large 2; huge. Eta squared and Omega squared: 0.01; small; 0.06 medium, 0.14 large

Table S4: Primers used for qPCR in this study

Gene name	Forward Primer (3′-5)	Reverse Primer (3′-5′)
Tnf-α1	GGC TGC CCC GAC TAT GTG	CTC CTG GTA TGA AGT GGC AAA TC
Tgf-β	GAG GTG ACC TGG GCA CCA T	GGC CAT GAG GAG CAG GAA
α-Sma	GAC CCT GAA GTA TCC GAT AGA ACA	CAC GCG AAG CTC GTT ATA GAA G
Vegf-R2	CTG CCT ACC TCA CCT GTT TCC	CGG CTC TTT CGC TTA CTG TTC
18S rRNA	GTA ACC CGT TGA ACC CCA TT	CCA TCC AAT CGG TAG TAG CG

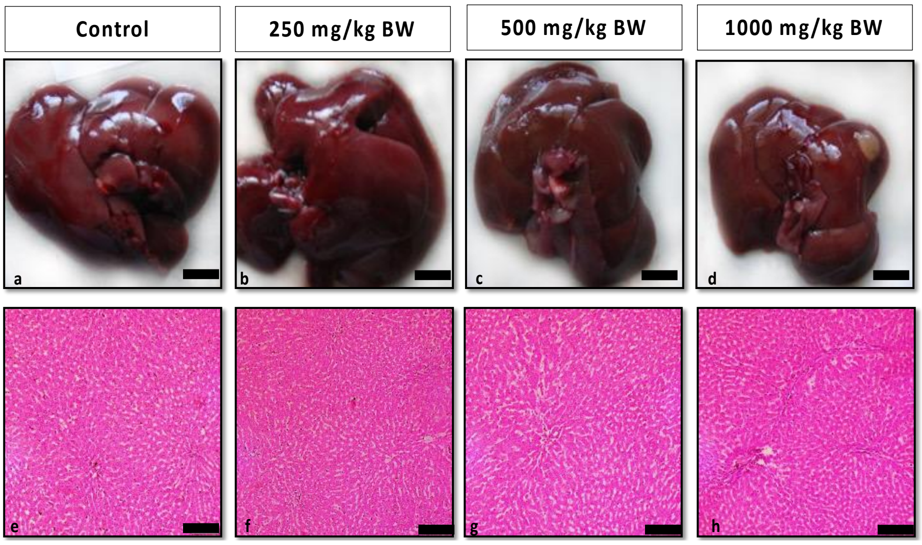

Figure S1: Administration of crude leaf suspension itself does not show any liver toxicities

Appearance of the control and different doses of CLS; 250 mg/kg body weight, 500 mg/kg body weight, 1000 mg/kg body weight, treated rat livers are shown. (a) Control group showed normal liver appearance. (b & c) 250 mg/kg body weight and 500 mg/kg body weight treated groups showed smooth liver surface similar to control group. (d) 1000 mg/kg body weight treated group showed small fat droplets in surface which were not prominent. Representative photographs are shown in scale bar = 10 mm. Histopathology by Hematoxylin and Eosin (H & E) stained liver sections of control and different doses of CLS; 250 mg/kg body weight, 500 mg/kg body weight and 1000 mg/kg body weight treated rat livers are shown. (e) Control group showed normal liver architecture. (f) 250 mg/kg body weight and (g) 500 mg/kg body weight treated groups showed normal hepatic architecture similar to control group. (h) 1000 mg/kg body weight showed initiation of ballooning degeneration. Representative photographs are shown in scale bar = 100 µm.
